# Does the Brain Matter? Cortical Alterations in Pediatric Bipolar Disorder: A Critical Review of Structural and Functional Magnetic Resonance Studies

**DOI:** 10.2174/1570159X20666220927114417

**Published:** 2023-05-12

**Authors:** Mario Luciano, Matteo Di Vincenzo, Emiliana Mancuso, Niccolò Marafioti, Arcangelo Di Cerbo, Vincenzo Giallonardo, Gaia Sampogna, Andrea Fiorillo

**Affiliations:** 1 Department of Psychiatry, University of Campania “L. Vanvitelli”, Naples, Italy

**Keywords:** Pediatric bipolar disorder, functional magnetic resonance, structural magnetic resonance, brain, functional connectivity, MRI

## Abstract

Pediatric bipolar disorder (PBD) is associated with significant psychosocial impairment, high use of mental health services and a high number of relapses and hospitalization. Neuroimaging techniques provide the opportunity to study the neurodevelopmental processes underlying PBD, helping to identify the endophenotypic markers of illness and early biological markers of PBD. The aim of the study is to review available studies assessing structural and functional brain correlates associated with PBD. PubMed, ISI Web of Knowledge and PsychINFO databases have been searched. Studies were included if they enrolled patients aged 0-18 years with a main diagnosis of PBD according to ICD or DSM made by a mental health professional, adopted structural and/or functional magnetic resonance as the main neuroimaging method, were written in English and included a comparison with healthy subjects. Of the 400 identified articles, 46 papers were included. Patients with PBD present functional and anatomic alterations in structures normally affecting regulations and cognition. Structural neuroimaging revealed a significant reduction in gray matter, with cortical thinning in bilateral frontal, parietal and occipital cortices. Functional neuroimaging studies reported a reduced engagement of the frontolimbic and hyperactivation of the frontostriatal circuitry. Available studies on brain connectivity in PBD patients potentially indicate less efficient connections between regions involved in cognitive and emotional functions. A greater functional definition of alteration in brain functioning of PBD patients will be useful to set up a developmentally sensitive targeted pharmacological and non-pharmacological intervention.

## 1 INTRODUCTION

The conceptualization of pediatric bipolar disorder (PBD) has seen an increasing interest in the last decades as a consequence of the awareness that its onset in children and adolescent is not so rare as previously thought. Findings report that the prevalence of PBD is 1% in youth aged 14-18 years and an additional 5.7% of youth show subsyndromal symptoms of BD [1]. Childhood onset of BD is very severe as it significantly affects patients’ ability to function normally during fundamental developmental stages [2]. In fact, the childhood onset of BD is associated with greater psychosocial impairment, higher use of mental health services and higher number of relapses and hospitalization compared to adult-onset [3], as happens with other mental disorders with an onset in childhood [4, 5]. Moreover, PBD usually follows a chronic/continuous course, with rapid mood cycles [6], prevalent irritability and high energy mixed with depressed mood. Moreover, patients with PBD often show impairments in several neurocognitive domains, including attention, working and verbal memory, executive functions, as well as deficits in social cognition, including impaired facial emotional recognition and emotional processing [7]. From the treatment perspective, patients with PBD often show treatment resistance, with increased metabolic side effects [8], which further worsens the long term-outcome and reduces recovery opportunities [9]. Additionally, PBD has higher comorbidity rates compared to adult-onset bipolar disorder. Conduct disorders, oppositional defiant disorders, attention deficit hyperactivity disorder (ADHD) and anxiety disorders often occur simultaneously with PBD, and frequently show overlapping symptoms with PBD, contributing to the diagnostic difficulties in PBD [10-12]. Over the past years, the suggestion that bipolar disorder (BD) may present differently in children compared to adults has reshaped the research in the field, reinforcing the idea that PBD may represent a distinct or partially separated clinical entity from adult-onset BD or that PBD is a phenotypically distinct subtype of BD [13].

Recently, novel neuroimaging techniques have allowed studying the neurodevelopmental processes underlying PBD, helping to identify the endophenotypic markers of illness and early biological markers of PBD without confounders, such as the long-term use of pharmacological treatments, illness duration, repeated illness episodes, heterogeneity of samples, and substance abuse [14]. Moreover, neuroimaging techniques, including anatomic, functional and biochemical imaging, can be useful for exploring similarities and differences between early- and late-onset BD [13], as well as other mental disorders, which can have their onset during childhood [15]. In particular, structural and functional magnetic resonance imaging (MRI) can be used in young patients without too much harm since they do not expose children to ionizing radiations or radioactive isotopes while providing a non-invasive *in vivo* definition of brain anatomy and functionality.

Despite this, MRI studies have been conducted mainly on adults with BD [16], since MRI techniques in children may exhibit difficulties in obtaining adequate scans or in performing reliable tasks during functional MRI. Moreover, given the higher occurrence of very rapid cycling, it is also difficult to co-ordinate scans with a specific mood state.

Available MRI studies carried out on adults with BP have consistently revealed spatial abnormalities of resting state networks functional connectivity, in particular in the medial prefrontal cortex and the anterior cingulated cortex with limbic-striatal structures, and in the regional homogeneity of the default mode network [17, 18], with a differential pattern of activation in the nucleus caudate, left temporopolar area (BA38), superior temporal gyrus (BA41/22), left cuneus (BA18) and left middle temporal gyrus in those presenting with psychotic symptoms [19].

Magnetic resonance studies can be grouped into those adopting structural or functional MR. Structural magnetic resonance imaging assesses the anatomic components of the brain, and it relies on differences in relaxation rates of water in different brain regions to generate image contrast [20]. It can be performed with or without a contrast agent, such as gadolinium, which improves soft tissue imaging capability. Conversely, functional MRI (fMRI) uses small signal changes in brain images to detect focal changes in blood flow. Images are usually captured in resting and stimulated states [13]. Theoretically, increased blood flow implies increased neuronal activity. This method is particularly relevant for the *in vivo* assessment of psychiatric disorders, since it is usually coupled with cognitive or affective tasks during scanning (*i.e*., colour naming Stroop task, visuospatial working memory tasks or international affective picture system tasks).

The last decades have witnessed a number of neuroimaging studies to be carried out in children with PBD. However, this large number of studies has led to heterogeneous findings. This may be due to the poor diagnostic definition of pediatric bipolar disorder [21], different adopted methodologies, and the involvement of different age groups, considering that structural and functional abnormalities may change according to the different developmental ages. Additionally, with regard to studies adopting fMRI task-dependent, heterogeneity may also be due to the different tasks adopted during the acquisition of scans.

Taken together, available data suggest the presence of structural and functional brain alterations in children with PBD. In particular, structural MRI studies have found white matter hyperintensities in both cortical and subcortical regions [1]. Likewise, white matter (WM) microstructural variations can be detected throughout diffusion tensor imaging (DTI), a specific MRI extension able to measure the anisotropic diffusion of molecules of water in *in vivo* cerebral tissue [22]. However, variables, such as gender, history of illness, duration, mood state, severity and presence of psychotic symptoms, make neuroimaging findings uneven and contrasting [23]. Functional MRI studies showed a decreased activity in fronto-striatal circuits in PBD adolescents with prefrontal subcortical activation [24, 25].

In this paper, we reviewed available studies on the assessment of structural and functional brain correlates associated with PBD. The already available reviews included only a few studies, especially on fMRI [1, 13, 14]. The increasing number of studies recently published has suggested the need for a re-examination of the literature. Both structural and functional MRI studies in patients with paediatric bipolar disorder have been included in the analysis.

## 2 METHODS

The relevant PubMed, ISI Web of Knowledge and PsychINFO databases have been searched for papers published until March 3, 2022, using keywords “Pediatric bipolar disorder” and “PBD” matched with “magnetic resonance”, “MRI”, “neuroimaging”. Studies were included in the review if they: 1) enrolled patients aged 0-18 years with the main diagnosis of paediatric bipolar disorder according to ICD or DSM made by a mental health professional; 2) adopted structural and/or functional magnetic resonance as main neuroimaging method; 3) were written in English; 4) included a control group of healthy subjects matched per age and gender; 5) excluded patients with PBD in comorbidity with any other neurodevelopmental disorder. Studies including other subsamples of patients (*i.e*., patients with ADHD) were included in the review only if it was possible to extrapolate from the results data on PBD *vs*. controls. Only randomized controlled trials, clinical trials, pilot studies and cohort studies were considered eligible for the review process.

In addition, reference lists of all included studies and relevant existing systematic reviews were checked for possible studies. After removing duplicates, full reports of potentially relevant studies were obtained, and two authors independently extracted content from the papers.

Entering the keywords in the relevant databases, 400 papers were identified; 140 were duplicates and excluded. After reading the abstracts, 214 additional papers were eliminated because they did not meet the inclusion criteria. Therefore, our review consisted of 46 papers.

Data on study design, sample characteristics, age range of recruited patients, characteristics of the neuroimaging method, psychopathological and psychosocial assessment, study aims, study design and main outcome were extracted independently by two authors (MDV and EM); discrepancies were resolved by discussion with a senior expert (ML). A narrative synthesis was undertaken due to the substantial methodological heterogeneity among studies. In particular, all studies included in the review were analysed in order to group them into categories, and the main results have been synthetized. According to the narrative synthesis, MDV, EM separately identified three groups: 1) structural MRI studies; 2) functional MRI studies, and 3) functional connectivity studies. After the second round of analysis and discussion with a senior expert (ML), studies were finally grouped into two broad categories: 1) studies using structural MRI (N=21), and 2) studies using functional MRI (M=25). Studies included in the latter grouping were further divided into studies analysing resting state (N=11) and those using a cognitive or affective task (N=14). The flowchart is shown in Fig. (**1**).

### 2.1 Quality Assessment According to the GRADE Criteria

The quality of quantitative studies was evaluated according to the GRADE criteria by two authors (MDV and EM) independently. Four levels of quality were identified from “high” to “very low”. The quality of evidence was considered “high”, when the study was a randomized controlled trial, and “low” in the case of observational studies. The attribution of a lower level was based on the presence of limitations, bias, inconsistencies, indirectness, and impreciseness in the study.

The authors independently assessed the studies against these criteria, and a discrepancy rate of about 10% was found, which was solved through discussion.

## 3 RESULTS

### 3.1 Structural MRI Studies

The present review identified 21 studies that have explored brain structural alterations in PBD patients. Half of the studies (11 out of 21) adopted a 3.0 Tesla scanner, and included very small sample sizes, ranging from 35 [26] to 201 PBD patients (of which 78 were diagnosed with “severe mood dysregulation” disorder [27]). All studies adopted a cross-sectional design, with the exception of Gogtay *et al.* [26] and Adleman *et al.* [27], which adopted a prospective methodology. Studies included in this section are reported in Table **1**.

Given the heterogeneity of results, and in order to improve the clarity of this section, results were grouped per area in terms of structural changes and alterations in bipolar youth’s brains.

### 3.2 Cortical Structures

Cerebral cortex exerts control on cognitive domains, such as attention, executive functioning, working memory and declarative memory, representing a primary focus in the research of structural correlates of PBD. Frazier *et al.* [28] have been among the first to study, by means of structural neuroimaging and parcellation methods, the cortical gray matter of lobes and gyri in a sample of 32 patients. They found a smaller bilateral parietal lobe (PL) and left temporal lobe (TL), with a subsequently reduced volume in bilateral PL postcentral gyrus, in left superior temporal and fusiform gyri. Moreover, they found a bilateral increased volume of parahippocampal gyri of the TL. Furthermore, the right middle frontal gyrus (which represents about 60% of the dorsolateral prefrontal cortex) was found of a significantly smaller volume, despite that volume alterations were not found in frontal lobes globally between patients and controls. In a combined neuropsychological and high-resolution structural MRI study, in which 15 psychotic PBD patients were matched with twenty healthy controls, James *et al.* [29] detected a reduction of grey matter density in the left occipital cortex, right occipital fusiform gyrus, right crus of the cerebellum, paracingulate, left inferior frontal gyrus/operculum, left frontal orbital cortex, and left angular gyrus.

Moreover, Simonetti *et al.* [30] reported a cortical thinning in frontal (right rostral anterior cingulate, right caudal anterior cingulate, right lateral orbitofrontal, right medial orbitofrontal), parietal (left and right inferior parietal, left posterior cingulate, left and right supramarginal) and occipital (left lingual) regions, belonging to the affective, frontoparietal and cingulo-opercular networks. Thinning of these cortical areas was correlated with higher levels of anger and lower levels of hostility. As regards cortical thickness, Cabeen *et al.* [31] highlighted that compared to controls, PBD patients show different maturation trajectories, with a greater cortical thinning with age in the left frontal pole. With respect to changes in brain structure over time in PBD patients, Gogtay *et al.* [26], despite a gradual increase in gray matter over time in temporal gyri and left ventrolateral prefrontal cortex (VLPFC), including left orbitofrontal cortex (OFC), found a diminished gray matter in the right OFC, bilateral anterior and subgenual cingulate and left posterior cingulate cortices.

Using voxel-based morphometry (VBM), a neuroimaging technique aimed at studying only gray matter volume without the contribution of non-brain and non-gray matter tissue, Wilke *et al.* [32] reported localized deficits in gray matter in the medial temporal lobe, anterior cingulate and orbitofrontal cortex. Contrary to Wilke *et al.* [32], Xiao *et al.* [33] reported an increased volume in orbitofrontal cortex on the left side. Another VBM study, simultaneously assessing multiple regions of the brain and avoiding bias due to manual tracing, reported a significant decrease of gray matter in the left dorsolateral prefrontal cortex (DLPFC), particularly in the Broadman area 9 [34]. Similarly, Adelman *et al.* [27] reported a reduced DLPF volume, whilst longitudinally, they reported that children with BD presented an abnormal increase in volume over time in the right superior and inferior parietal lobule (IPL) and in the bilateral precuneus.

Baloch *et al.* [35] and Sanches *et al.* [36] found no significant differences in the subgenual prefrontal cortex (SGPFC), involved in emotional regulation and reward processes due to strong bidirectional connections with the amygdala, hippocampus and parahippocampus, in PBD patients compared to controls. According to Baloch *et al.* [35], PBD patients with one or more first-degree relatives affected by any mood disorder presented a significantly lower volume in left SGPFC when compared to non-familial groups and healthy controls.

Lastly, Fernandes *et al.* [37] explored the global structural cortical connectivity in PBD patients, in which the presence of psychotic symptoms seems to relate to widespread changes in structural brain connectivity and network topology, particularly in the orbitofrontal cortex, frontal gyrus, amygdala, hippocampus and basal ganglia.

### 3.3 Ventricles

Only one study [38] was specifically designed to assess structural changes of cerebral ventricles in PBD patients. The authors found that PBD patients with manic symptoms presented increased volumes in the bilateral ventricles, third ventricles and whole ventricles, while euthymic patients had enlarged volumes in the third, fourth and whole ventricles.

### 3.4 Amygdala and Hippocampus

Amygdala is involved in affective, cognitive and vegetative functions [39]. Adopting the VBM methodology, Dickstein *et al.* [34] and Xiao *et al.* [33] detected a significantly decreased volume in the left amygdala. In a cross-sectional study, Cui *et al.* [40] used automated neuroanatomical quantification in order to assess the amygdala and its sub-nuclei, reporting that PBD patients in manic state presented diminished volumes in whole bilateral amygdala, basal nucleus, cortical-amygdaloid transition and accessory basal nucleus, while euthymic PBD patients showed volume loss in the bilateral accessory basal nucleus and left cortical-amygdaloid transition.

Studies included in this review [23, 33, 40, 41] consistently found a significantly reduced hippocampal volume. Focusing specifically on mania, Gao *et al.* [22] found that PBD patients presented a significantly decreased volume in the left hippocampus. The same structural findings were reported in manic and euthymic pediatric subjects by Xiao *et al.* [33].

In a more recent Chinese cross-sectional study involving 28 PBD patients with past or current psychotic symptoms, 26 PBD patients without psychotic symptoms and 19 healthy controls, Gao *et al.* [23] found decreased gray matter volumes in the bilateral amygdala-hippocampus-parahippocampal complex.

### 3.5 Basal Ganglia

Wilke *et al.* [32] reported that basal ganglia, including the anterior putamen and the head of caudate, have been found to be bilaterally larger in BD-I patients (currently in the manic or mixed state) than healthy controls, while Dickstein *et al.* [34] found diminished volume in nucleus accumbens (NA), which is thought to be involved in reward mechanisms. On the contrary, Ahn *et al.* [42] found a larger right NA, and Adleman *et al.* [27] an increased globus pallidus (GP) volume.

### 3.6 White Matter (WM)

WM integrity can be detected in DTI studies, which analyse the fractional anisotropy (FA) as means of WM fiber density, axonal diameter, and myelination; lower FA values can be correlated with inflammation, oedema, gliosis or demyelination. Loss of integrity of WM, as well as lower values of FA, appear to be involved in the pathophysiology of PBD. FA is significantly lower in the right anterior cingulate region when PBD patients are in a current manic state [22]. Similar results were detected in studies focused on corona radiata [43] and anterior commissure [44], whose FA values were negatively correlated with lifetime aggression. In a multi-modal MRI study by Cabeen *et al.* [31], PBD patients showed decreased FA (bilaterally) and axial diffusivity with age in superficial and deep WM in frontal, temporal and striatal regions. Furthermore, the same study found a widespread effect in the corpus callosum, a pivotal structure in the interhemispheric connection whose diminished values of FA have been observed by James *et al.* [29] and Saxena *et al.* [44]. Contrary to these studies, no significant differences in WM microstructure were found by Teixera *et al.* [45] when they compared 18 unmedicated PBD patients with as many offspring children of bipolar parents and twenty healthy controls.

### 3.7 Functional MRI Studies

25 studies have been included in this section. All studies had a cross-sectional design except for one longitudinal study [46]. All included studies adopted a 3.0 Tesla scanner, and the studied population ranged from 20 [43, 47] to 80 [48] subjects. To improve clarity of reports, studies were grouped into those adopting an fMRI resting state (N=11) and task-dependent studies (N=14) (Table **2**).

### 3.8 Resting-state fMRI Studies

Zhang *et al.* [49], assessing the alterations in the prefrontal-limbic-subcortical circuit and the prefrontal-striatal-thalamic (PFST) loops, which are thought to account for processing external emotional stimuli and internally generated emotion and integrating emotional and cognitive output to modulate behaviour [20], reported an increased resting state functional connectivity in PBD type I group between left GP and right caudate (CAU), and between right CAU and left inferior frontal gyrus (IFG), and a decreased connectivity between right NA and left superior frontal gyrus (SFG), and between right GP and right thalamus. Moreover, in PBD type II patients, the same authors found a decreased connectivity between right GP and right thalamus, between right CAU and left middle frontal gyrus (MFG), and between right NA and left GP. Striatal abnormalities were also reported by Lu *et al.* [50] in a resting state fMRI study employing the Amplitude of Low-Frequency Fluctuations (ALFF), which measures signal magnitude on a voxel-by-voxel basis. They found increased ALFF in the caudate and pallidum as well as decreased ALFF in the precuneus, superior parietal lobule and inferior occipital gyrus. Moreover, Guo *et al.* [51] reported decreased functional connectivity between orbital-frontal gyrus and amygdala, between left superior frontal gyrus and left putamen, and between superior frontal gyrus and insula. Similar results have been reported by Gao *et al.* [52], who highlighted a decreased Regional Homogeneity (ReHo) in the medial frontal gyrus, middle frontal gyrus and putamen. ReHo alterations were also reported by Xiao *et al.* [53], who found an increased ReHo in the hippocampus, anterior cingulate cortex, parahippocampal gyrus and left caudate, as well as decreased ReHo in the precuneus, precentral gyrus, superior parietal lobe, orbitofrontal cortex and superior temporal gyrus.

Alterations in functional connectivity in cognitive circuits have also been reported. In particular, Dickstein *et al.* [54] found that compared to controls, PBD patients have reduced functional connectivity between the left DLPFC and the right superior temporal gyrus (STG), and between the STG and frontal, striatal, and parahippocampal areas. These circuits are all involved in working and declarative memory.

Alterations in both affective and cognitive circuits were reported by Xiao *et al.* [55] and Gao *et al.* [19]. In particular, Xiao *et al.* [51] found that manic PBD patients present reduced cortical ReHo signal in the superior temporal gyrus, the superior parietal lobe, and precentral gyrus, and altered subcortical ReHo signal in the insula and cerebellum crus, while euthymic group presented reduced cortical ReHo signal in the STG and superior parietal lobule. Gao and colleagues [56] investigated differences between PBD patients with and without psychotic symptoms by using four-dimensional (spatiotemporal) consistency of local neural activities (FOCA) to investigate the local spontaneous brain activity by integrating the temporal and spatial information of local brain regions. Interestingly, they found that, compared to the control group, the PBD group with psychotic symptoms showed decreased FOCA in the left supplementary motor area and superior frontal gyrus, and showed increased FOCA in the triangular inferior frontal gyrus. In contrast, the PBD group without psychotic symptoms exhibited decreased FOCA in the superior occipital gyrus and postcentral gyrus and increased FOCA in the orbital inferior frontal gyrus.

Lastly, alterations in functional connectivity in thalamo-frontal loops seem to be involved in the pathophysiology of PBD patients. In fact, Guo *et al.* [57] found a significantly reduced functional connectivity between the bilateral thalamus and middle frontal gyrus MFG in manic PBD patients. Moreover, globally, PBD exhibited a reduction in grey matter cortical thickness especially in left middle frontal gyrus and in the bilateral superior frontal gyrus.

Recent studies identified aberrant functional connectivity in distributed, large-scale, resting-state networks [58]. Among intrinsic connectivity networks, Calhoun *et al.* [59] found abnormalities in cortical networks, such as the default mode network (DMN), executive network (EN), and salience network (SN) in adults with BD. The default mode network appears to be involved in introspection, theory of mind, and internally directed cognition, whereas salience network plays a role in the segregation of important stimuli and in modulating responses in the sensory, motor, and association cortices [60]. Moreover, the EN network appears to be active primarily during tasks requiring attention to internal stimuli [60]. Lopez-Larson *et al.* [48] reported increased internetwork functional connectivity between DMN and SN, while Cao *et al.* [61] found altered integration within the same networks in euthymic PBD. Furthermore, they also detected abnormalities in global functional connectivity density: increased in anterior insula and reduced in the temporoparietal junction, the angular gyrus and the occipital lobule.

### 3.9 Task-related fMRI Studies

The frontolimbic circuit, primarily consisting of the orbitofrontal cortex, ventrolateral prefrontal cortex (VLPFC), the anterior cingulate cortex, insula and limbic regions, including amygdala and hippocampus, is known as the brain’s affective network, underlying the processing and regulation of emotions. Several task-related fMRI studies have explored alterations in this neuronal pathway, highlighting its crucial role in PBD physiopathology. Passarotti *et al.* [62] examined abnormal regional functional connectivity (FC) in brain networks during an affective working memory task; in response to emotional *vs*. neutral stimuli, PBD patients showed altered FC in frontolimbic circuit (decreased in amygdala and VLPFC and increased in the medial prefrontal cortex). In a previous fMRI emotional task-related study, the same authors [63] reported that the PBD group showed greater activation in the right and left medial prefrontal cortex, VLPFC and cingulate regions (dorsal ACC, posterior cingulate gyrus), bilateral precuneus, right supramarginal gyrus and left middle temporal gyrus.

Wegbreit *et al.* [64] found an impaired functional integration of the frontolimbic network when participants viewed negative valenced words in a colour matching task, in terms of increased functional connectivity in bilateral amygdala, parahippocampal gyrus, hippocampus, inferior VLPFC, orbitofrontal cortex, anterior insula, superior temporal pole, and the inferior cerebellar vermis, and of decreased functional connectivity in the superior vermis and bilateral thalamus. Wegbreit and colleagues [64] argued that these alterations result in global impaired recruitment of the entire frontolimbic affective circuit of PBD patients, compared to controls, while performing an affective task. Similarly, Garrett *et al.* [65] assessed amygdala abnormalities in PBD, reporting greater amygdala activation and DLPFC hypoactivation during perception of facial expression in emotional tasks.

Affective circuit dysfunction in PBD patients was also probed by Pavuluri *et al.* [66] by using an emotion-processing task, which elicited greater activation in the amygdala, insula, middle frontal gyrus and left posterior cingulate cortex than controls in incidental conditions. Moreover, they reported a hypoactivation of the prefrontal cortex and pregenual anterior cingulate cortex in these patients. Previously, Pavuluri *et al.* [67] also reported that in negative affect condition (*i.e*., when negatively valenced words are presented), patients display a hyperactivation in the anterior cingulate cortex (ACC) and amygdala, and hypoactivation of cortical regions, such as VLPFC and DLPFC.

One noteworthy ability impaired in PBD is face recognition and processing. PBD patients show face emotion misinterpretation, as reported by Rich *et al.* [68], in a faces fMRI task. In fact, they reported a significantly reduced connectivity between amygdala and posterior cingulate/precuneus and fusiform gyrus/parahyppocampal gyrus in PBD patients.

Apart from the affective circuit, other brain pathways are altered in PBD patients. The frontostriatal circuits are neural pathways connecting frontal lobe regions, mainly DLPC and VLPC, with the basal ganglia (*i.e*., dorsolateral caudate and ventral striatum) that mediate the motor, cognitive, and behavioral functions. Two fMRI studies using emotional go/ nogo task [69, 70] explored the cognitive circuits in PBD patients and found hyperactivation in cognitive circuits in both manic and euthymic patients. The main involved regions were DLPFC, inferior parietal lobule, superior/middle frontal gyrus, superior/middle temporal gyrus, insula, posterior cingulate gyrus and posterior cerebellum lobe. Nelson *et al.* [71] reported a DLPFC hyperactivation in PBD while assessing neural responses on a simple motor response flexibility task.

Furthermore, PBD patients present behavioural deficits on reversal learning tasks. In reversal learning, the individual first learns to make a discrimination, such as choosing a black object in a black-white discrimination problem and then is supposed to learn to reverse his choice (*i.e*., to choose the white object). Dickstein *et al.* [54], analysing the neural correlates of reversal learning deficits, reported that PBD patients show significantly greater neural activity than controls in the dorsomedial frontal cortex, lateral superior frontal cortex and parietal cortex during the reversal phase, particularly in response to punished reversal error. These deficits account for dysfunction among PBD patients in regions associated with processing response conflict, implementing alternative responses, and controlling attention. Chang *et al.* [24], adopting a visuospatial working memory task to indagate DLPFC, ACC and subcortical areas, reported that compared to controls, PBD patients show greater activation in ACC, putamen, thalamus, DLPFC and inferior frontal gyrus under task conditions.

Additionally, assuming that response inhibition processes rely on frontostriatal circuits, Passarotti *et al.* [72] reported that PBD subjects show deficits in prefrontal areas, such as VLPFC and DLPFC, inferior and middle frontal gyri and ACC, and increased activity in bilateral caudate while performing response inhibition task. This result, which is in line with emerging findings, highlights an impairment in PBD patients lying at the interface between cognitive and affective brain circuits that are responsible for combined deficits in executive function and mood stability. Such interface of affective VLPFC-amygdala circuitry and cognitive DLPFC-striatal circuitry in PBD was investigated by Yang *et al.* [46] over a 3-year period. At baseline, they found greater activation in the emotional and cognitive processing regions implicated in the top-down modulation. These areas included the right DLPFC, amygdala, VLPFC, bilateral ventral striatum, and ACC. Additionally, Passarotti *et al.* [73] assessed how working memory circuits are affected by emotion processing in a sample of 23 PBD patients and 19 controls. The PBD group exhibited reduced activation in a left IFG region at the junction of DLPFC and VLPFC, which integrates cognitive and affective processes during working memory processes for the angry *versus* neutral face condition.

### 3.10 Summary of Design Quality

The GRADE criteria were adopted to evaluate the quality of included studies. The majority of the studies (N = 42, 95.4%) were of moderate quality, being cross-sectional studies. The remaining two papers were rated as low quality due to the small sample size. Of note, given the topic of the included studies, the RCT design could not be adopted, and therefore, none of the studies could be evaluated as “high” quality according to GRADE criteria, according to which all observational studies have to be rated “low” and could be upgraded only to “moderate” score, in case there is a low risk of bias in reporting results or confounding factors have been assessed appropriately.

## 4 DISCUSSION

The adoption of structural and functional MR technology in pediatric patients with bipolar disorder is fundamental in order to understand the neurobiology of bipolar disorder and to understand changes in early brain structure and function that could constitute early markers for the development of a full-blown disorder in adulthood. Research on the neuropathophysiology of PBD is expanding rapidly. Unfortunately, the increasing interest in the neuropathophysiology of PBD has not corresponded to a better understanding of the neural bases of this disorder. Available studies report very heterogeneous results, probably due to the differences in adopted methodology. In fact, several studies have adopted a different MRI methodology (*i.e*., 1, 5 Tesla *vs*. 3.0 Tesla), which are not readily comparable. Moreover, as regards fMRI studies, the adoption of heterogeneous tasks has led to very heterogeneous results. Another factor that limits comparisons among available studies lies in the different phases of the disorders in which patients have been recruited. In fact, while the majority of studies have recruited patients in the euthymic state, several others have recruited patients in manic, mixed or depressed phases, and some other studies have recruited only PBD patients with psychotic symptoms regardless of the phase of the disorder. This is a major confounding factor, especially when comparing functional MRI task-dependent studies, since the presence of manic or depressed symptoms is associated with various alterations in functional connectivity, particularly in the affective circuitry [73, 74].

Taken together, the results of our review suggest that patients with pediatric bipolar disorders present functional and anatomic alterations in structures normally affecting regulations and cognition [75]. Structural neuroimaging has revealed a significant reduction in gray matter, with cortical thinning in bilateral frontal, parietal and occipital cortices, particularly in the dorsolateral and ventrolateral prefrontal cortex and subgenual prefrontal cortex. Although not all studies have reported consistent results, these findings are in line with the hypothesis that PBD is associated with multiple deficits in the whole frontal cortex. Interestingly, these alterations have been rarely reported in adults with bipolar disorder, considering that this region usually undergoes substantial developmental changes during adolescence [76, 77]. Therefore, a reduction in volumes of different areas of the prefrontal cortex could be considered an early proxy for the presence of PBD. Moreover, anatomical MRI reported a reduction in the hippocampus, which is correlated with impairments in declarative memory and emotion regulation shown by PBD subjects [78]. With regard to emotional regulation, the hippocampus is involved in stress management and emotional regulation. Hippocampus also plays a role in the regulation of cognitive function, in particular in the memory preservation and visual-spatial orientation [79].

Several subcortical nuclei have been found to be reduced in volume, including the amygdala, whose main function is related to vigilance and emotional regulation. Alterations in amygdala volume were associated with illness duration and disease progression in PBD [80]. It is worth noting that similar alterations have not been found in young patients with schizophrenia [81-84], and therefore, these alterations can be considered more specific for PBD.

Particularly relevant is the finding of a reduced fractional anisotropy in the white matter of PBD patients, both in corona radiata and in corpus callosum. Corona radiata is a fiber pathway containing descending and ascending axons that carry important projections between the frontal cortex and the brainstem. It is associated with the cortico-pontine, cortico-bulbar and spinal tracts, and targets basal ganglia along these pathways. Corpus callosum provides interhemispheric connections between the left and right prefrontal cortices, integrates inter-hemispheric functioning and plays a role in the integration of emotional and cognitive information [85]. All these alterations are thought to underlie emotional dysfunctions, particularly irritability, which is characteristic of early-onset bipolar disorder.

One could argue that anatomical structural brain alterations do not necessarily reflect alterations in functions. For this reason, more recently, functional MRI has been more frequently used to assess brain structures. This method allows the detection of all neural circuits alterations also when brain structures are anatomically indistinguishable from healthy controls. Additionally, fMRI allows detecting patterns in brain activation while patients perform cognitive or affective tasks, thus providing information about the pathophysiology of mental disorders. The majority of fMRI studies in PBD used cognitive or affective tasks with the aim to explore alterations in functional connectivity in affective modulation, cognitive control and interaction between affective and cognitive circuitries.

With only a few exceptions, those studies reported a reduced engagement of the frontolimbic circuitry during affective tasks, with increased activation of the amygdala and a reduction in the VLPC activity. Since amygdala is involved in attributing emotional significance to everyday experiences, its hyperactivity can be interpreted with a pattern of excessive emotional arousal in the limbic subcortical structures, coupled with a failure in the top-down control, usually operated by the prefrontal cortex. Amygdala hyperactivity is consistent with a clinical pattern of increased emotional reactivity, usually seen in patients with PBD.

While assessing the functional connectivity of cognitive circuitry in PBD patients, a global hyperactivation of cortical and subcortical structures (including DLPC, ACC, putamen and thalamus) has been detected. These alterations could reflect inefficient recruitment of these regions to achieve satisfactory task performance or could reflect compensatory activity in response to dysfunction in other brain areas. However, alterations in these areas account for reduced cognitive flexibility and an inefficient functioning of a series of regions implicated in attention control and response selection [54]. Impairment in cognitive functioning could have implications in clinical practice, including a reduced response rate to psychosocial interventions [86-89], which mainly rely on cognitive skills and poor academic performances.

## CONCLUSION

In conclusion, available studies on brain connectivity in PBD patients potentially indicate less efficient connections between regions involved in cognitive and emotional functions. However, despite an increasing number of studies, several questions remain unanswered. In fact, future studies should better investigate whether alterations in brain anatomical structures and functions are phases-specific or constitute a stable neural correlate of paediatric bipolar disorder; a more in-depth assessment of correlations between clinical picture and brain correlates is advisable. In fact, available evidence does not support the idea that the differences in the clinical presentation of bipolar disorders between children/adolescence and adults are due to different alterations of brain circuitries. Moreover, poor evidence exists about the interaction between cognitive and affective circuitries in PBD patients [90]. Lastly, the identification of clear neural markers to identify people at high risk to have a poor clinical outcome would be useful [91, 92]. Moreover, the effect of early treatment on brain volumes should be assessed. In fact, only few studies have assessed the role of mood stabilizer in children with PBD on brain volumes and changes in brain activation [93]. Available evidence suggests a potential neuroprotective role of valproate [94] and lithium [95]. However, available evidence on this issue is very scarce, and replication studies with larger sample sizes are highly needed.

A greater functional definition of alteration in brain functioning of PBD patients will be useful to set up a developmentally sensitive targeted pharmacological and non-pharmacological intervention, which will ideally help children and adolescents with bipolar disorder to mitigate the long-term complications of the disorder, including poor academic performance and poor social functioning, and to reduce treatment resistance in these patients.

## Figures and Tables

**Fig. (1):**
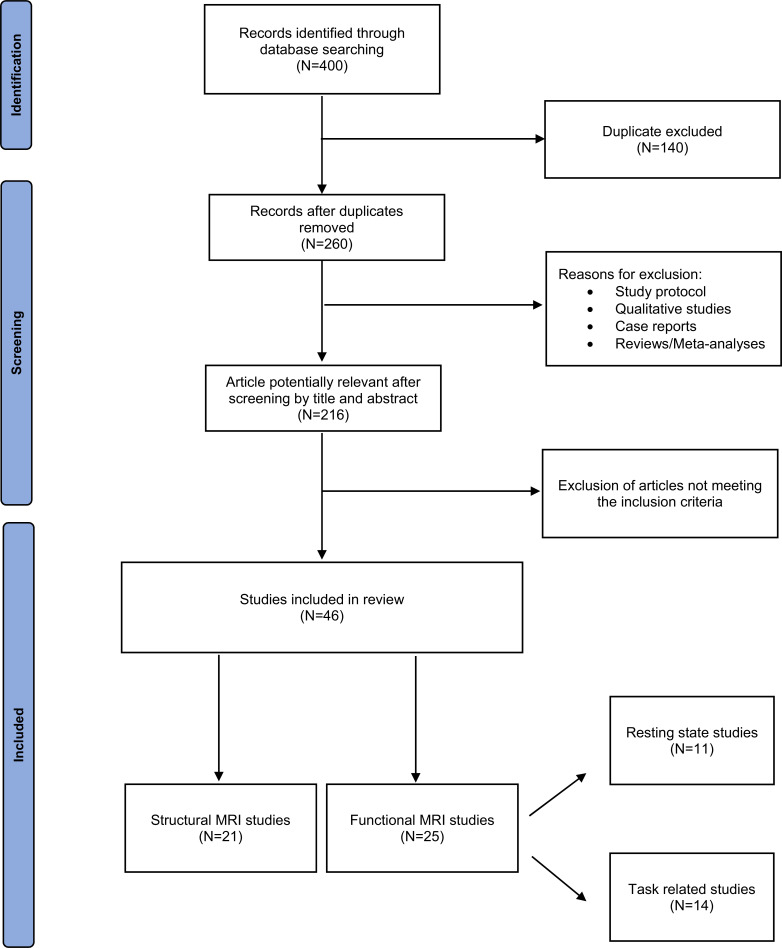
PRISMA flow diagram of selection of studies for inclusion in the review.

**Table 1:** Structural MRI studies.

**Study and Country**	**Sample**	**Subject Age (M ± SD)**	**MR Method**	**Diagnosis**	**Study Aims**	**Study ** **Design**	**Main Results**
[22] Gao *et al*. (2013), China	18 manic PBD patients 18 healthy controls	15.1 ± 1.8 14.1 ± 1.6	3.0 Tesla	DSM-IV	To assess brain structural changes in manic bipolar children and adolescents.	Cross-sectional	Decreased GMV in the left hippocampus and lower FA in the right anterior cingulate were found in manic PBD patients.
[23] Gao *et al*. (2021), China	28 PBD patients with psychotic symptoms 26 PBD patients without psychotic symptoms 19 healthy controls	15.4 ± 1.6 14.7 ± 2.0 14.2 ± 1.6	3.0 Tesla	DSM-IV	To investigate brain structural alterations in PBD patients with and without psychotic symptoms.	Cross-sectional	Decreased GMV in the bilateral amygdala, hippocampus, parahippocampal complex, left superior temporal gyrus, left inferior frontal gyrus, bilateral putamen and left precentral gyrus was found in PBD patients with psychotic symptoms, whereas PBD patients without psychotic symptoms showed decreased GMV in the left inferior frontal gyrus, left precentral gyrus, left supramarginal gyrus, and right inferior parietal lobule.
[26] Gogtay *et al*. (2007), USA	9 BPD 8 multidimensionally impaired (psychotic broad phenotype) 18 healthy controls	13.3 ± 3.7 (first scan) 12.1 ± 2.5 (first scan) 13.8 ± 3.3 (first scan)	1.5 Tesla	DSM-IV	To conduct the first prospective study of cortical brain development in PBD before and after the illness onset.	Longitudinal	An increase over follow-up in the gray matter on the anterior and middle portions of the middle and inferior temporal gyri and in the middle portion of the superior temporal gyrus in BPD patients. Differences in gray matter volume in the left VLPFC as well as an overall loss in gray matter volume in the right orbitofrontal cortex. On the medial cortical surface, bilateral gray matter loss in anterior and subgenual cingulate cortices, most prominently in the subgenual regions, in PBD patients.
[27] Adleman *et al*. (2012), USA	55 PBD patients (mood state NR) 78 patients with severe mood dysregulation 68 healthy controls	12.7 ± 2.4 14.2 ± 2.6 13.9 ± 2.3	1.5 Tesla	DSM-IV	To explore structural differences in association cortices and basal ganglia between PBD patients, individuals with severe mood dysregulation and healthy controls.	Cross-sectional and longitudinal	Both PBD patients and those with severe mood dysregulation showed reduced volume in pre-SMA, insula, DLPFC, and globus pallidus. Longitudinal analyses showed an abnormal increase in volume in the superior/inferior parietal lobule over time in children with PBD, compared to healthy controls and those with severe mood dysregulation.
[28] Frazier *et al*. (2005b), USA	32 PBD patients (16 with mixed, 6 with manic, 3 with depressive episode; 7 euthymic) 15 healthy controls	11.2 ± 2.8 11.2 ± 3.0	1.5 Tesla	DSM-IV	To assess alterations in cortical volumes in PBD patient and to determine if they show a specific pattern of cortical gray matter deficits.	Cross-sectional	Smaller bilateral parietal lobe and left temporal lobe, with significantly smaller volume in bilateral postcentral gyrus, left superior temporal and fusiform gyri and right middle frontal gyrus in PBD patients compared to healthy controls.
[29] James *et al*. (2011), UK	15 PBD patients with psychotic symptoms 20 healthy controls	15.0 ± 2.0 15.3 ± 1.0	1.5 Tesla	DSM-IV-TR	To detect structural brain changes in PBD subjects with psychotic symptoms (delusions and/or hallucinations).	Cross-sectional	Reduced FA in the anterior half of the corpus callosum was found in the PBD group. Reduced gray matter density was detected in the left occipital cortex, right occipital fusiform gyrus, right crus of the cerebellum, paracingulate, left inferior frontal gyrus ⁄operculum, left frontal orbital cortex and left angular gyrus.
[30] Simonetti *et al*. (2021), Italy and USA	23 PBD patients (mood episode NR) 23 healthy controls	12.3 ± 3.2 12.0 ± 3.3	3.0 Tesla	DSM- 5	To investigate the relationship between cortical thickness and impulsiveness in PBD patients.	Cross-sectional	PBD patients showed cortical thinning in prefrontal and parietal cortices. This structural finding was associated with higher levels of anger and lower levels of hostility.
[31] Cabeen *et al*. (2018), USA	26 PBD (2 with hypomanic, 3 with depressive and 3 with the mixed episode; 18 euthymic) patients 26 healthy controls	13.9 ± 2.6 13.9 ± 3.0	3.0 Tesla	DSM-IV-TR	To assess multimodal structural imaging biomarkers of development trajectories in pediatric bipolar disorder.	Cross-sectional	The rate of cortical thinning with age was greater in patients in the left frontal pole. In both superficial and deep white matter of frontotemporal striatal regions, healthy controls showed higher FA and axial diffusivity with age, whilst PBD patients an opposite trend.
[32] Wilke *et al*. (2004), USA	10 PBD patients (6 with mixed, 4 with manic episode) 52 healthy controls	14.5 ± 1.8 14.5 ± 1.3	3.0 Tesla	DSM-IV	To assess brain morphometry differences between bipolar youth and controls by using volumetric and voxel-based approaches.	Cross-sectional	Significantly greater gray matter volume in PBD patients in the left temporal lobe and, bilaterally, in the thalamus and basal ganglia. The latter finding was confirmed by voxel-based morphometry. In the same group, localized deficits of gray matter were found in the medial temporal lobe, orbitofrontal cortex and anterior cingulate.
[33] Xiao *et al*. (2020), China	21 manic PBD patients 19 euthymic PBD patients 18 healthy controls	15.0 ± 1.8 15.1 ± 1.9 14.1 ± 1.6	3.0 Tesla	DSM-5	To investigate the differences in brain structures between children with PBD patients and controls.	Cross-sectional	Reduced volume in the left hippocampus, parahippocampal gyrus and amygdala and increased volume of the left orbitofrontal cortex were found in both manic and euthymic groups.
[34] Dickstein *et al*. (2005), USA	20 PBD patients (mood state not specified) 20 healthy controls	13.4 ± 2.5 13.3 ± 2.3	1.5 Tesla	DSM-IV	To determine brain volume alterations in children with BPD using voxel-based morphometry.	Cross-sectional	Reduced gray matter volume in the left DLPFC of PBD patients. With a less conservative statistical threshold, gray matter reductions were detected in the left accumbens and left amygdala.
[35] Baloch *et al*. (2010), USA	51 PBD patients (mood state NR) 41 healthy controls	13.2 ± 2.9 13.7 ± 2.7	1.5 Tesla	DSM-IV	To assess the volume of SGPFC in subjects with PBD.	Cross-sectional	SGPFC volumes were not significantly different between PBD and controls samples. However, PBD subjects who had one or more first-degree relatives with mood disorders showed significantly smaller left hemisphere SGPFC.
[36] Sanches *et al*. (2005), USA	15 PBD patients (2 with the depressive episode; 13 euthymic) 21 healthy controls	15.5±3.5 16.9±3.8	1.5 Tesla	DSM-IV	To compare SGPFC volumes in children and adolescents with BD and healthy controls.	Cross-sectional	No statistically significant differences between patients and healthy controls with respect to right or left SGPFC volumes.
[37] Fernandes *et al*. (2019), UK	15 PBD patients with psychotic symptoms 15 healthy controls	15.0 ± 2.0 15.7 ± 1.3	1.5 Tesla	DSM-IV-TR	To compare network properties of whole-brain structural connectomes of euthymic PBD patients with psychosis and healthy controls.	Cross-sectional	Changes in the structural connectivity of PBD patients with psychotic symptoms in both cortical and subcortical networks, including the orbitofrontal cortex, frontal gyrus, amygdala, hippocampus and basal ganglia, were found.
[38] Kuang *et al*. (2020), China	20 manic PBD patients 21 euthymic PBD patients 19 healthy controls	14.5 ± NR 15.0 ± NR 15.0 ± NR	3.0 Tesla	DSM-IV	To assess ventricular volumetric alterations in manic and euthymic PBD patients and their correlations with cognitive changes.	Cross-sectional	Enlarged volumes in the bilateral, third ventricle and whole ventricles, were found in PBD subjects in the manic phase. Euthymic PBD patients displayed increased volumes in the third ventricle, fourth ventricle and whole ventricles.
[40] Cui *et al*. (2020), China	20 manic PBD patients 20 euthymic PBD patients 19 healthy controls	15.3 ± 1.8 15.6 ± 1.6 14.4 ± 1.3	3.0 Tesla	DSM-5	To compare amygdala and subnuclei volumes among manic, euthymic PBD patients and healthy controls.	Cross-sectional	Manic PBD patients exhibited significantly decreased volumes in the bilateral amygdala and its basal nucleus, cortico-amygdaloid transition and accessory basal nucleus. Euthymic patients had decreased volume in the accessory basal nucleus and left cortico-amygdaloid transition.
[41] Frazier *et al*. (2005c) USA	43 PBD patients (22 with mixed, 7 with manic and 5 with the depressive episode; 9 euthymic) 20 healthy controls	11.3 ± 2.7 11.0 ± 2.6	1.5 Tesla	DSM-IV	To evaluate brain volumes in early-onset bipolar disorder cases.	Cross-sectional	Smaller hippocampal volumes in PBD patients, mainly females. Significantly smaller cerebral volumes in both male and female PBD patients.
[42] Ahn *et al*. (2007), USA	46 PBD patients (24 with mixed, 8 with manic and 5 with the depressive episode; 9 euthymic) 22 healthy controls	11.3 ± 2.7 11.1 ± 2.7	1.5 Tesla	DSM-IV	To investigate the basal ganglia in PBD patients.	Cross-sectional	Larger right nucleus accumbens volume was detected in PBD youth, mostly in prepubertal age. No significant asymmetry or volume differences were found with regard to caudate, putamen and globus pallidus.
[43] Pavuluri *et al*. (2009), USA	13 PBD patients (mood state not specified) 15 healthy controls	14.8 ± 2.5 13.7 ± 2.7	3.0 Tesla	DSM-IV	To investigate white matter integrity in PBD.	Cross-sectional	Significantly lower FA was observed in anterior corona radiata in PBD patients compared to healthy controls.
[44] Saxena *et al*. (2012), USA	10 PBD patients (1 with manic, 1 with mixed state and 1 with depressive episode; 7 euthymic) 10 healthy controls	13.9 ± 3.6 13.6 ± 3.6	3.0 Tesla DTI	DSM-IV	To investigate alterations of corpus callosum and anterior commissure in aggressive PBD youth.	Cross-sectional	Lower FA values in the callosal genu and anterior commissure were found in PBD patients. Moreover, FA values in the anterior commissure were negatively related to a lifetime history of aggression.
[45] Teixera *et al*. (2014), Brazil	18 PBD patients (6 with manic and 4 with mixed episode; 8 euthymic) 18 healthy offspring 20 healthy controls	12.3 ± 2.8 12.7 ± 3.1 12.7 ± 2.6	3.0 Tesla DTI	DSM-IV-TR	To assess the presence of white matter alterations in healthy children at risk, as well as in pediatric, non-medicated bipolar patients.	Cross-sectional	No significant differences in white matter microstructure between PBD patients, healthy offspring and healthy controls were found.

**Abbreviations:** PBD - pediatric bipolar disorder; ADHD - attention/deficit hyperactivity disorder; GMV - gray matter volume; FA - fractional anisotropy; pre-SMA - pre-supplementary motor area; DLPFC - dorsolateral prefrontal cortex; DTI - diffusion tensor imaging; SGPFC - subgenual prefrontal cortex; VLPFC - ventrolateral prefrontal cortex; NR - not reported.

**Table 2:** Functional MRI studies.

**Study and Country**	**Sample**	**Subject Age ** **(M ± SD)**	**Method**	**Task**	**Diagnostic Tool**	**Study Aims**	**Study Design**	**Main Results**
**Resting State Studies**								
[48] Lopez-Larson *et al*. (2017), USA	32 euthymic PBD patients 48 healthy controls	15.1 ± 2.0 14.5 ± 2.4	3.0 Tesla	-	DSM-IV-TR	To investigate how brain connectivity may be altered in PBD patients.	Cross-sectional	Increased functional connectivity in PBD patients has been found between the default mode network (involved in introspection, theory of mind, and internally directed cognition) and salience networks (involved in the detection and segregation of important stimuli from insignificant stimuli, and to modulate responses in the sensory, motor, and association cortices).
[49] Zhang *et al*. (2022), China	36 PBD-I patients (mood episode NR) 22 PBD-II patients (mood episode NR) 19 healthy controls	15.3 ± 1.9 14.6 ± 1.5 14.1 ± 1.6	3.0 Tesla	-	DSM-IV	To identify morphometric differences in PBD-I and PBD-II; to explore altered resting state functional connectivity patterns across the corticolimbic network.	Cross-sectional	Volume reduction of the right pallidum in PBD-I patients, an abrupted prefrontal-striatal-thalamic functional connectivity in PBD-I group and decreased functional connectivity in PBD-II relative to HCs and PBD-I.
[50] Lu *et al*. (2014), China	18 manic PBD patients 18 healthy controls	15.1 ± 1.8 14.1 ± 1.6	3.0 Tesla	-	DSM-IV-TR	To explore the spontaneous neuronal activity in resting state in PBD patients.	Cross-sectional	PBD patients presented increased ALFF in bilateral caudate and left pallidum as well as decreased ALFF in left precuneus, left superior parietal lobule, and bilateral inferior occipital gyrus.
[51] Guo *et al*. (2021a), China	16 euthymic PBD patients 16 healthy controls	15.1 ± 1.7 14.1 ± 1.5	3.0 Tesla	-	DSM- IV	To evaluate cognitive damage and alteration of consistency of temporal-spatial spontaneous resting-state activity of euthymic PBD subjects.	Cross-sectional	Decreased functional connectivity between right orbital frontal gyrus and left amygdala, between left superior frontal gyrus and left putamen, and between left superior frontal gyrus and left insula. Increased functional connectivity between the right superior occipital gyrus and right hippocampus.
[52] Gao *et al*. (2014), China	17 depressed PBD patients 18 healthy controls	14.4 ± 1.8 14.1 ± 1.6	3.0 Tesla	-	DSM-IV	To detect abnormalities of baseline brain functionality in depressed PBD patients.	Cross-sectional	Decreased regional homogeneity in the medial frontal gyrus, bilateral middle frontal gyrus and middle temporal gyrus, and the right putamen in the PBD group.
[53] Xiao *et al*. (2013), China	15 manic PBD patients 15 healthy controls	15.0 ± 1.7 14.1 ± 1.5	3.0 Tesla	-	DSM-IV-TR	Assess resting-state brain activity to explore neurobiological biomarkers of the disorder.	Cross-sectional	PBD patients showed a significant increase in regional homogeneity in the bilateral hippocampus, the right anterior cingulate cortex, the right parahippocampal gyrus, and the left caudate. Regions with decreased ReHo included the bilateral precuneus, the bilateral precentral gyrus, the bilateral superior frontal gyrus, the bilateral superior parietal lobe, the right orbitofrontal cortex, and the right superior temporal gyrus.
[54] Dickstein *et al*. (2010), USA	15 PBD euthymic patients 15 healthy controls	13.7 ± 3.3 14.0 ± 3.1	3.0 Tesla	-	DSM-IV-TR	To assess the neural underpinnings of pediatric BD.	Cross-sectional	PBD patients had significantly greater negative resting state functional connectivity between the left DLPFC and the right superior temporal gyrus.
[55] Xiao *et al*., (2019), China	21 muthymic PBD patients 22 manic PBD patients 19 healthy controls	14.9 ± 1.7 15.1 ± 1.8 14.2 ± 1.6	3.0 Tesla	-	DSM-5-TR	Assess abnormalities in PBD in the resting state.	Cross-sectional	Reduced cortical regional homogeneity in the STG, the superior parietal lobe, precentral gyrus, and altered subcortical regional homogeneity signal in the insula and cerebellum crus I in manic PBD. Reduced cortical regional homogeneity signal in the STG and superior parietal lobe in euthymic PBD.
[56] Gao *et al*. (2021), China	27 PBD patients with psychotic symptoms 25 non-psychotic PBD patients 19 healthy controls	15.4 ± 1.6 14.8 ± 1.9 14.2 ± 1.6	3.0 Tesla	-	DSM- IV	To investigate the brain functional differences between PBD patients with and without psychotic symptoms.	Cross-sectional	Significant among-group differences in consistency of local neural activities in the left triangular inferior frontal gyrus, left supplementary motor area, left precentral gyrus, right postcentral gyrus, right superior occipital gyrus, and right superior frontal gyrus.
[57] Guo *et al*. (2021b), China	40 PBD patients (20 with the manic episode; 20 euthymic) 19 healthy controls	15.3 ± 1.7 14.2 ± 1.6	3.0 Tesla	-	DSM-IV	To assess structural and functional alterations of the thalamo-frontal loop among the different mood states in PBD.	Cross-sectional	Thalamo-frontal hyperconnectivity with middle frontal gyrus in manic PBD, and thalamo-frontal hypoconnectivity with precentral gyrus in euthymic PBD.
[61] Cao *et al*. (2020), China	16 euthymic PBD patients 16 healthy controls	15.1 ± 1.7 14.1 ± 1.5	3.0 Tesla	-	DSM-IV	To investigate functional connectivity changes in euthymic PBD patients.	Cross-sectional	PBD patients exhibited greater global functional connectivity density in the left anterior insula and lower global functional connectivity density in the right temporoparietal junction, the left angular gyrus, and the bilateral occipital lobule.
**Task-dependent Studies**								
[24] Chang *et al*. (2004), USA	12 PBD patients (affective mood episode NR) 10 healthy controls	14.7 ± 3.0 14.4 ± 3.2	3.0 Tesla	Visuospatial Working Memory Task IAPS Task	DSM IV	To examine possible abnormalities in the DLPFC and anterior cingulate cortex, as well as selected subcortical areas.	Cross-sectional	Greater activation in the bilateral anterior cingulate cortex, left putamen, left thalamus, left DLPFC, and right inferior frontal gyrus was found in PBD patients. In viewing negatively valenced pictures, subjects with PBD had greater activation in the bilateral DLPFC, inferior frontal gyrus, and right insula. For positively valenced pictures, subjects with PBD had greater activation in the bilateral caudate and thalamus, left middle/superior frontal gyrus, and left anterior cingulate cortex.
[46] Yang *et al*. (2013), USA	13 PBD patients (8 with the manic episode; 5 euthymic) 10 healthy controls	13.3 ± 2.3 13.6 ± 3.0	3.0 Tesla	Pediatric affective color matching task	DSM IV	To examine activation changes at the interface of affective and cognitive systems over a 3-year period in PBD subjects.	Longitudinal	At baseline, in response to emotional *vs*. neutral words, patients with PBD showed greater activation in the right DLPFC, amygdala, VLPFC, bilateral anterior cingulate cortex, and ventral striatum. After pharmacological treatment, increased activation in DLPFC in the PBD group normalized by 16 weeks. By 3 years, normalization was observed in VLPFC, ACC, amygdala, and striatum.
[62] Passarotti *et al*. (2012), USA	41 PBD patients 16 healthy controls	14.0 ± 2.3 14.6 ± 3.2	3.0 Tesla	Working memory task	DSM-IV	To examine whether adolescents with PBD have abnormal regional functional connectivity in distributed brain networks during an affective working memory task.	Cross-sectional	PBD showed decreased functional connectivity in regions involved in emotion processing (right amygdala) and in emotion regulation regions (right VLPFC), while functional connectivity was increased in emotion evaluation regions (bilateral MPFC). PBD exhibited greater connectivity in left DLPFC, caudate, and right VLPFC.
[63] Passarotti *et al*. (2010a), USA	17 PBD patients (12 with manic and 5 with mixed episode) 14 healthy controls	14.3 ± 2.0 14.1 ± 2.4	3.0 Tesla	Emotional Valence Stroop Task	DSM IV	To investigate the neural bases of cognitive control of emotion processing in PBD.	Cross-sectional	PBD group exhibited greater activation in VLPFC and anterior cingulate cortex relative to controls. During cognitive control of emotion processing, PBD patients deployed the VLPFC to a greater extent than controls.
[64] Wegbreit *et al*. (2011), USA	34 manic PBD patients 14 healthy controls	13.4 ± 2.3 14.2 ± 3.1	3.0 Tesla	Color-matching task	DSM-IV	To assess functional connectivity among PBD patients who are responders to pharmacotherapy and those who are non-responders.	Cross-sectional	Impaired functional integration in PBD patients in frontolimbic network was found when participants viewed negatively valenced words. PBD medication responders showed greater connectivity of the amygdala into the network before and after treatment compared to non-responders.
[65] Garrett *et al*. (2012), USA	20 euthymic PBD patients 21 healthy controls	15.6 ± 2.1 15.4 ± 2.7	3.0 Tesla	Emotional/ facial expressions stimuli and task	DSM-IV	To assess amygdala/ DLPFC abnormalities in PBD.	Cross-sectional	PBD patients exhibited less DLPFC and greater amygdala activation during the perception of facial expressions.
[66] Pavuluri *et al*. (2009), USA	10 euthymic PBD patients 10 healthy controls	15.2 ± 2.0 14.3 ± 2.1	3.0 Tesla	Incidental and Directed Emotion Processing Task	DSM-IV	To use fMRI to probe the affective circuitry dysfunctions underlying disturbances in emotion processing and emotional reactivity in PBD.	Cross-sectional	The incidental condition (*i.e*., when patients had to judge whether the presented face was older or younger than 35 years) elicited greater activation in the right amygdala and the right insula, the left middle frontal gyrus, and the left posterior cingulate cortex in patients with PBD. In both incidental and directed (*i.e*., when patients had to judge whether the presented faces’ affect was positive/happy or negative/angry) conditions, patients with PBD showed less activation in the right prefrontal cortex (superior, middle, and inferior frontal gyri) and the pregenual anterior cingulate cortex.
[67] Pavuluri *et al*. (2008), USA	10 euthymic PBD patients 10 healthy controls	15.0 ± 2.4 16.2 ± 1.3	3.0 Tesla	Color-matching task	DSM IV	To assess the functional integrity of attentional control and affect processing circuitry in PBD patients.	Cross-sectional	In the negative affect condition, relative to the neutral condition, patients with bipolar disorder demonstrated greater activation of bilateral pregenual anterior cingulate cortex and left amygdala, and less activation in right rostral VLPFC and DLPFC.
[68] Rich *et al*. (2008), USA	33 PBD patients (11 with hypomanic, 2 with depressive and 1 with mixed episode; 18 euthymic) 24 healthy controls	14.4 ± 3.0 14.4 ± 2.2	3.0 Tesla	Faces fMRI task	DSM IV	To elucidate the neural mechanisms associated with PBD face emotion misinterpretation.	Cross-sectional	PBD subjects had significantly reduced connectivity between the left amygdala and two regions: right posterior cingulate/precuneus and right fusiform gyrus/parahippocampal gyrus. Deficits were evident regardless of mood states.
[69] Xiao *et al*. (2021a), China	18 euthymic PBD patients 16 manic PBD patients 17 healthy controls	14.9 ± 1.8 15.1 ± 1.6 14.2 ± 1.6	3.0 Tesla	Emotional Go/No-go task	DSM-5	To explore the differences in neural activities between manic and euthymic PBD during emotional response inhibition.	Cross sectional	Both manic and euthymic PBD subjects showed increased activities in the cognitive and emotional regulation circuits compared to healthy individuals. Manic PBD patients exhibited increased activities in the left superior frontal gyrus. Hyperactivity in the left superior frontal, left middle frontal, and right inferior frontal gyrus in manic PBD was positively associated with false response errors.
[70] Xiao *et al*. (2021b), China	18 euthymic PBD patients 17 healthy controls	15.2 ± 1.6 14.2 ± 1.6	3.0 Tesla	Emotional go/no-go fMRI tasks	DSM-5	To compare differences in the activities of emotional and cognitive circuits between euthymic PBD and controls subjects.	Cross-sectional	Euthymic PBD patients showed increased activities in the DLPFC, inferior parietal lobule, superior/middle frontal gyrus, superior/middle temporal gyrus, insula, posterior cingulate gyrus and posterior cerebellum lobe relative to healthy controls.
[71] Nelson *et al*. (2007), USA	25 PBD patients (5 with a hypomanic episode and 20 euthymic) 17 healthy controls	13.4 ± 2.5 14.6 ± 1.8	3.0 Tesla	Visual attention and response flexibility task	DSM-IV	To determine if neuronal responses differed between PBD and control subjects on a simple motor response flexibility task.	Cross-sectional	PBD patients generated significantly more activity in the left DLPFC and in the primary motor cortex than healthy controls.
[72] Passarotti *et al*. (2014), USA	15 PBD (10 with manic and 5 with mixed episode) 15 healthy controls	13.2 ± 2.6 14.1 ± 3.2	3.0 Tesla	Response inhibition task	DSM-IV	To identify similarities and differences in the neural substrate of response inhibition deficits that are associated with these disorders.	Cross-sectional	For the Stop *versus* Go condition, PBD group showed less activation in the right medial and left middle frontal, left inferior/middle frontal gyri and in the right pregenual ACC and greater activation in the left superior temporal gyrus and inferior parietal lobule and right posterior cingulate cortex.
[73] Passarotti *et al*. (2010b), USA	23 PBD patients (affective episode NR) 19 healthy controls	13.5 ± 2.5 13.4 ± 2.5	3.0 Tesla	n-Back task with emotional faces	DSM-IV	To assess how working memory circuits are affected by face emotion processing in PBD.	Cross-sectional	PBD patients showed greater activation in right medial prefrontal cortex, left DLPFC, left posterior cingulate, striatal regions, bilateral thalamus, bilateral parietal regions and right fusiform gyrus.

**Abbreviations**: DLPFC - dorsolateral prefrontal cortex; STG - superior temporal gyrus; VLPC - ventrolateral prefrontal cortex; IAPS International Affective Picture System; MPFC - medial prefrontal cortex; PBD - Pediatric Bipolar Disorder; NR - Not reported; ALFF - amplitude of low-frequency fluctuation; ACC - anterior cingulate cortex; fMRI - functional magnetic resonance imaging.

## LIST OF ABBREVIATIONS

ACC: Anterior Cingulate Cortex
ADHD: Attention Deficit Hyperactivity Disorder
BD: Bipolar Disorder
DMN: Default Mode Network
DTI: Diffusion Tensor Imaging
EN: Executive Network
FA: Fractional Anisotropy
GP: Globus Pallidus
IPL: Inferior Parietal Lobule
MFG: Middle Frontal Gyrus
MRI: Magnetic Resonance Imaging
NA: Nucleus Accumbens
OFC: Left Orbitofrontal Cortex
PBD: Paediatric Bipolar Disorder
PL: Parietal Lobe
SFG: Superior Frontal Gyrus
SN: Salience Network
STG: Superior Temporal Gyrus
TL: Temporal Lobe
VBM: Voxel-based Morphometry
VLPFC: Ventrolateral Prefrontal Cortex
